# Allied health – 3002. Aero-allergen sensitivity among patients suffering from bronchial asthma in Bangalore, Karnataka, India

**DOI:** 10.1186/1939-4551-6-S1-P179

**Published:** 2013-04-23

**Authors:** Giriyanna Gowda, Chitra Nagaraj, BG Parasuramalu, - Huliraj

**Affiliations:** 1 Community Medicine, Kims Hospital & Research Center, Allergy Clinic, Bangalore, India; 2 TB & Chest Disease, Kims, Bangalore, India

## Background

Asthma is a serious public health problem throughout the world. The prevalence of asthma has increased in last two to three decades possibly due to change in indoor and outdoor environment. Allergens are one of the many factors which trigger an attack of asthma. Skin prick test is useful in identifying the offending allergen in bronchial asthma.

## Methods

The study was conducted at allergy center, Kempegowda Institute of Medical Sciences (KIMS) Hospital & Research center, Bangalore from January 2011 – December 2011. Skin Prick Test was done in 139 patients suffering from bronchial asthma diagnosed based on GINA guidelines. Skin Prick Test was performed using 49 allergens extracts after taking informed consent from the patients. Allergen extracts included 19 pollens, 10 fungi, 5 dusts, 2 dust mites (Dermatophagoides farinae and Dermatophagoides pteronyssinus), 10 insects and 3 epithelia.

## Results

Out of 139 patients who underwent skin prick test, 40% (56) were males and 60% (83) were females. Majority i e 60% were in the age group of 21 -40 years. 43% (60) had family history of asthma/atopy. 80 % (111) had allergic rhinitis, 24% (34) had chronic urticaria and 24% (33) had allergic conjunctivitis. Out of 139 patients 100 (71.94%) were sensitive for one or more allergens. The common offending allergens found in the study were dust mites (DF and DP) - 49.28%, dusts - 7.2%, pollens - 6.77%, insects - 6.62%, fungi - 4.53%, epithelia - 1.92%.Comparison of asthmatics between those with positive skin prick test and negative skin prick test was done. Those with positive skin prick test had age of onset < 20 years and family history of asthma compared to negative skin prick test patients and found statistically significant (p<0.05).

## Conclusions

The most common allergens in bronchial asthma were dust mites followed by dusts and pollens. Identifying possible allergens in asthma patients help in allergen avoidance and immunotherapy in these patients.

